# Comorbidities in Canadian patients with hereditary angioedema: a quantitative survey study

**DOI:** 10.1186/s13223-025-00953-8

**Published:** 2025-03-19

**Authors:** Paul K. Keith, Gina Lacuesta, Dawn Goodyear, Stephen D. Betschel, Belinda Yap, Marie-France Dansereau, Nataly Tanios, Rami El-Sayegh, Maye Machnouk, Hachem Mahfouz, Adriana Martin, Susan Waserman

**Affiliations:** 1 Department of Medicine, McMaster University, 3V47 HSC 1280 Main St W, Hamilton, ON L8S 4K1 Canada; 2 Dalhousie University, Halifax, NS Canada; 3 Division of Hematology and Hematologic Malignancies, Department of Medicine, University of Calgary, Calgary, AB Canada; 4 Department of Clinical Immunology and Allergy, University of Toronto, Toronto, ON Canada; 5 St. Michael’s Hospital, Toronto, ON Canada; 6 Cencora, Innomar Strategies Inc, Oakville, ON Canada; 7 CSL Behring Canada Inc, Ottawa, ON Canada

**Keywords:** Hereditary Angioedema, Autoimmune, Allergy, Asthma, C1 inhibitor, Comorbidities

## Abstract

**Background:**

Evidence linking hereditary angioedema (HAE) to the potential association of developing other comorbidities, and how it is affected by HAE treatment is needed. The objective of this study is to identify comorbidities and measure the prevalence in HAE patients, compared to the prevalence in the general population using multiple Canadian sources when available.

**Methods:**

A quantitative survey design via a self-administered anonymous online questionnaire was conducted from October 13, 2022, to January 11, 2023. Respondents were individuals with HAE, enrolled in the CSL Behring patient support program (CSL Behring PLUS+; PSP).

**Results:**

This study included 123 patients (81% female; 60% HAE-1/HAE-2, 24% HAE Normal C1-INH (nC1-INH), 16% unsure of HAE type; 85% of patients were on long-term prophylaxis plus on-demand). Patients reported using the following HAE treatments: C1-esterase inhibitor (subcutaneous or intravenous), lanadelumab, icatibant, danazol, and tranexamic acid. Respondents (69%) reported at least one: autoimmune condition, asthma, or allergy. Reported autoimmune conditions (psoriasis, rheumatoid arthritis, inflammatory bowel disease, chronic urticaria, lupus, and psoriatic arthritis) were much higher than the general population (31% versus 5–8%). Patient-reported allergies were two times higher than the general population (54% versus 27%; i.e., aeroallergens) and asthma rates nearly two times higher than the general population (17% versus 8–11%).

**Conclusion:**

This cohort of HAE patients, most of whom were on prophylaxis, reported an increased prevalence of certain comorbidities compared to the general Canadian population. Healthcare professionals should be aware of the potentially increased risk of autoimmune conditions, allergies, and asthma in patients with HAE.

## Introduction

Hereditary angioedema (HAE) is a rare disease with an estimated prevalence of approximately 1.1 to 1.6 per 100,000 [1]. HAE is characterized by episodes of potentially debilitating and life threatening angioedema affecting the skin and mucous membranes [2]. These episodes are recurrent and unpredictable, accompanied by swelling, most frequently involving the face, airways (including potentially fatal laryngeal attacks), extremities, genitals, gastrointestinal tract, and mesenteric structures [3–5]. In addition, to these complications, researchers have questioned the prevalence of certain comorbidities in patients with HAE, including, but not limited to, autoimmune disorders and malignancies [6–8].

HAE can be categorized into three different types including HAE with deficit C1-inhibitor levels (HAE-1), HAE with dysfunctional C1-inhibitor (HAE-2), and HAE with normal C1-inhibitor function (HAE nC1-INH) [9]. C1-INH is the main regulator of the complement, contact and coagulation systems. The complement system plays a key role in the immune system’s ability to maintain tissue homeostasis and effectively clear soluble immune complexes and cell debris [10]. This results in the depletion of early complement components. C4 is an essential component of the complement system, and there is accumulating evidence that a complete or partial deficiency of C4 is associated with higher risk of symptoms following infections and autoimmune disorders such as rheumatoid arthritis (RA), systemic sclerosis, and systemic lupus erythematosus [6, 8, 10]. A population-based cohort study conducted by Björkman Sa et al. [7]. not only confirmed these results from previous studies [8, 11–15] but also demonstrated an increased risk of autoimmune diseases and other comorbidities, such as thromboembolic disease [7].

Therefore, the effects of C1-INH deficiency and its consequences are yet to be fully understood, and additional evidence linking HAE to an increased risk of developing autoimmune diseases, malignancies or other comorbidities is needed. The main objectives of this survey were to identify comorbidities and their prevalence in HAE patients, enrolled in a Patient Support Program (PSP), and to compare the reported prevalence of comorbidities with that in the general Canadian population. The secondary objectives of the survey were to examine (i) the severity of the reported comorbidities, (ii) the onset of comorbidities in relation to HAE treatment initiation, and (iii) the evolution of comorbidities throughout the HAE treatment course, (iv) family history and predisposition to comorbidities, and (v) hospital admissions history in relation to comorbidities.

## Methods

### Study design

This study was a quantitative survey design that used a self-administered anonymous online questionnaire, accessible only via a unique URL link through a personal invitation. The survey was conducted between October 13, 2022, to January 11, 2023, and the online questionnaire was hosted on Qualtrics XM, a subscription software platform for experience management [16]. The questionnaire was 63 questions that was estimated to take 20 to 40 min to complete, depending on the patient’s medical history.

The questionnaire, survey protocol and classification of diseases were developed in collaboration with a C1 working advisory committee consisting of 5 Canadian clinicians who treat HAE patients. The questionnaire mostly included close-ended questions to better normalize the input collected and better guide the patients through their responses. The questions explored patients’ HAE treatment journey, any diagnosed comorbidities (autoimmune diseases, chronic illnesses, malignancies, etc.), onset of symptom comorbidities in relationship to their HAE diagnosis and treatment, family history of reported comorbidities, all currently prescribed treatments, overall health, and basic demographics. Prevalence of reported concomitant conditions are compared with prevalence within the Canadian population obtained through secondary research. As sources vary, these were intended as indicators only.

### Participants and recruitment

Participants were eligible for the survey if they were diagnosed with HAE, 18 years of age or older, currently living in Canada, fluent in English (or French in the province of Quebec), able and willing to provide informed consent to participate in the survey, and currently enrolled in the CSL Behring PSP (CSL Behring PLUS+). The CSL Behring PLUS + program, managed and administered by Bayshore Healthcare, is a PSP that helps provide access to patients living with HAE to pdC1-INH (subcutaneous or intravenous). Innomar Strategies and Bayshore HealthCare collaborated to recruit eligible patients.

Participation in this survey was on a voluntary basis and respondents had the right to decline participation in the survey at any point. Patient consent was electronically obtained prior to the initiation of the online questionnaire. A nominal compensation was offered to all respondents, and information required for compensation was kept confidential by Innomar Strategies and rendered anonymous before any data analysis was conducted. The study was approved by the Health Ethics Research Board at Advarra; this study was carried out in accordance with the recommendations of the Health Ethics Research Board.

### Statistical analysis

Data was analyzed using WINCROSS DESKTOP^®^20 [17], an advanced cross-tabulation software. All data was analyzed with descriptive statistics and whenever appropriate or possible, depending on the final sample size, two-tailed statistical tests was performed to determine statistically significant differences in groups of interest such as patients’ age groups, HAE types, HAE treatments received, etc. The level of statistical significance was defined as *p* < 0.05.

Notably, open-ended questions were coded to identify common themes to develop a codebook and quantify the data obtained and then included in the analysis. These open-ended questions asked patients to report medical conditions and prescribed treatments that were not explored in the close-ended questions; thus, subjective interpretation of this qualitative data is limited.

## Results

### Demographic characteristics

The study included 123 patients with HAE (Table 1). The majority of patients were female (81%) compared to male (19%), and a mean age of 44.9 years (18–83; 14.4 standard deviation (SD)), with most patients being between 34 and 44 years of age (28%). The majority of patients reported HAE-1 (46%), followed by HAE nC1-INH (24%), and HAE-2 (14%); additionally, 16% of patients were unsure of the type of HAE they had. The mean ages of HAE symptom onset, HAE diagnosis, and HAE treatment initiation were 17.6 (15 SD), 29.7 (30 SD), and 32.4 (16 SD) years, respectively. Most respondents were diagnosed between 0 and 5 years after their first symptoms (47%). Many of these respondents began medication within the same year as diagnosis (56%), though 24% started treatment between 1 and 5 years after diagnosis, and 19% of respondents started treatment longer than 5 years after diagnosis.

**Table 1:** Demographic characteristics of study population

	Respondents (*n* = 123)
**Sex**	
Male	23 (18.7%)
Female	99 (80.5%)
Non-Binary	1 (0.8%)
**Age**	
18–24	7 (5.7%)
25–34	24 (19.5%)
35–44	35 (28.4%)
45–54	23 (18.7%)
55–64	24 (19.5%)
65–74	6 (4.9%)
75+	4 (3.3%)
Mean	44.9
Median	41.0
Range	18.0–83.0
SD	14.4
**Province**	
Atlantic Canada	10 (8.1%)
Ontario	33 (26.8%)
Quebec	23 (18.7%)
Western Canada	57 (46.3%)
**HAE Type**	
HAE-1	56 (45.5%)
HAE-2	17 (13.8%)
HAE nC1-INH	30 (24.4%)
Unsure	20 (16.3%)
**Treatment Type**	
Prophylaxis + On-demand	105 (85.3%)
On demand only	18 (14.6%)
**HAE Treatment Medication– Routine Prophylaxis** (*n* = **105)**	
Intravenous pdC1inh	32 (30.5%)
Danazol	1 (0.9%)
Subcutaneous pdC1inh	51 (48.6%)
Lanadelumab	14 (13.3%)
Tranexamic Acid	1 (0.9%)
Other	6 (5.7%)
**HAE Treatment Medication– On-Demand** (*n*** = 120)**	
Intravenous pdC1inh	75 (61.0%)
Icatibant	28 (22.8%)
Icatibant + intravenous pdC1NH	3 (2.4%)
Subcutaneous pdC1inh	13 (10.6%)
Other	1 (0.8%)
**Age of HAE Symptoms Onset**	
1–17	84 (68.3%)
18–24	15 (12.2%)
25–34	7 (5.7%)
35–44	6 (4.9%)
45–54	6 (4.9%)
55–64	4 (3.3%)
65–74	1 (0.8%)
75+	0 (0.0%)
Mean	17.6
Median	13.0
SD	15.0
**Age of HAE Diagnosis**	
1–17	31 (25.2%)
18–24	22 (17.9%)
25–34	27 (22.0%)
35–44	18 (14.6%)
45–54	11 (8.9%)
55–64	9 (7.3%)
65–74	3 (2.4%)
75+	2 (1.6%)
Mean	29.7
Median	26.0
SD	17.6
**Age of HAE Treatment Initiation**	
1–17	18 (14.6%)
18–24	26 (21.1%)
25–34	29 (23.6%)
35–44	22 (17.9%)
45–54	14 (11.4%)
55–64	9 (7.3%)
65–74	2 (1.6%)
75+	3 (2.4%)
Mean	32.4
Median	29.0
SD	16.2

Abbreviations: HAE (hereditary angioedema), HAE-1 (HAE with deficit C1-inhibitor levels), HAE-2 (HAE with dysfunctional C1-inhibitor), HAE nC1-INH (HAE with normal C1-inhibitor), pdC1inh (plasma-derived C1-INH), SD (standard deviation)

*Other prophylaxis treatment includes: icatibant and berotralstat

*Other on-demand treatment includes: icatibant, intravenous pdC1inh and icatibant (combo), subcutaneous pdC1inh 2000 UI

With regards to current treatment type, the majority of patients reported the use of long-term prophylaxis (LTP) plus on-demand (85%), and the remaining patients used on-demand only (15%). LTP therapy was taken by 89% of HAE-1 patients, 76% of HAE-2 patients, 80% of HAE nC1-INH, and 90% of patients uncertain of HAE type. Most respondents used subcutaneous pdC1inh for routine prophylaxis (49%). For on-demand treatment, the majority of respondents used intravenous pdC1inh (63%).

### Comorbidities

**Fig. 1:**
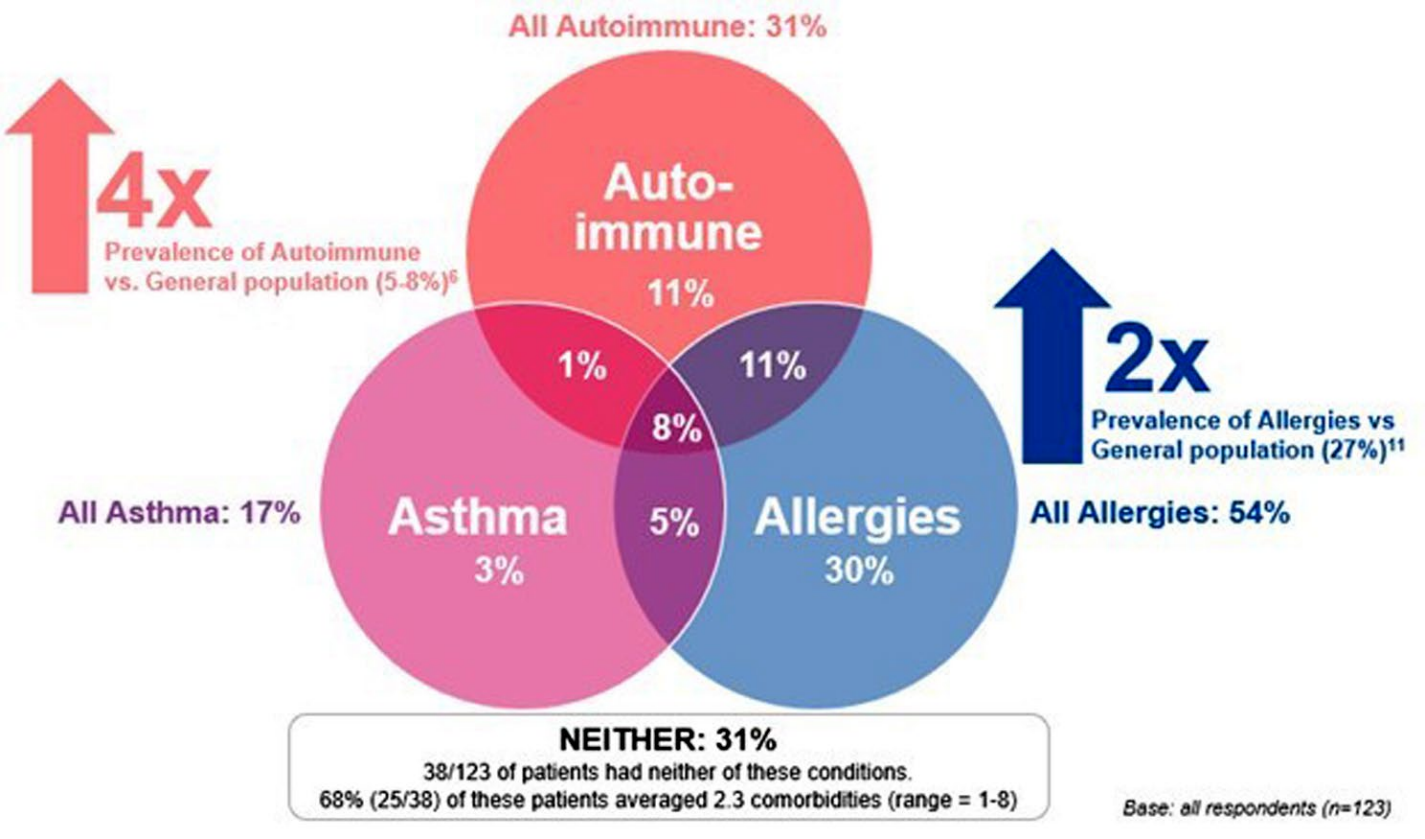
Overlap between autoimmune, allergies, and asthma

Overall, when grouped by International Classification of Diseases (ICD) class, the most common comorbidities reported were related to the respiratory system (e.g., allergies/asthma) at 39%, the immune system at 38%, and neurological systems (e.g., anxiety and depression) at 37%. HAE patients (69%) had at least one of the following conditions: autoimmune, asthma, and allergy. Autoimmune conditions were reported by 31% of patients, while 54% of patients reported allergies, and 17% of patients reported asthma. There is also overlap between these three conditions, with 29% of respondents with autoimmune conditions also reporting asthma, 61% reporting allergies, and 44% of allergy sufferers also reporting an autoimmune condition and/or asthma. 31% of patients did not report any of these three conditions (though they did report other comorbidities). Table 2; Fig. 1 illustrate the proportion of patients living with autoimmune, allergic, and asthma conditions and the proportion of patients living with only one, two, or all three of these conditions.

**Table 2:** Number of patients with autoimmune, allergies, and asthma

Conditions	Respondents, *n* (%) (*n* = 123)
**Patients with all 3 conditions (autoimmune, asthma, and allergies)**	**10 (8.1%)**
**Patients with 2 conditions**	**20 (16.2%)**
Autoimmune and asthma	1 (0.8%)
Autoimmune and allergies	13 (10.6%)
Asthma and allergies	6 (4.9%)
**One condition only**	
Autoimmune only	14 (11.4%)
Asthma only	4 (3.3%)
Allergies only	37 (30.1%)
**None**	**38 (30.1%)**
Overall*	
Total autoimmune	38 (30.9%)
Total asthma	21 (17.1%)
Total allergies	66 (53.7%)

*Overall percentages for each condition based on 123 respondents. Total tallies include double counting since some patients have multiple conditions

**Fig. 2:**
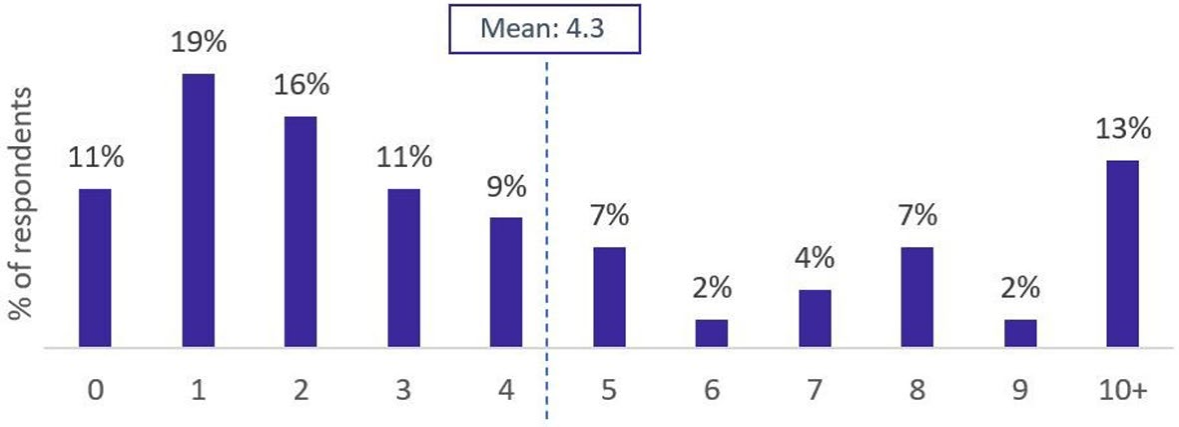
Number of reported comorbidities per patient

Patients reported living with 4.3 comorbidities on average, while some reported over 10 comorbid conditions. Patients most commonly had 1 comorbidity (19%), followed by 2 comorbidities (16%). Notably, 13% of patients had 10 + comorbidities. Figure 2 depicts the distribution of patients by number of comorbidities.

The most frequently reported comorbidities in the survey were allergies (e.g., medication, pollen, and pets/dander), depression, and hypertension. When compared to the general population, allergies were reported by 54% percent of respondents compared to 27%[18] in the general population, depression was reported in 28% of HAE patients compared to 21–25% [19] in the general population, and hypertension was reported in 24% of HAE patients which is similar to the general population (23% [20]). Prevalence of reported cancers were either similar to the general population or significantly lower as in the case of prostate, breast and skin cancer.

### Autoimmune disease

Approximately one-third of respondents reported having diseases that are classified as autoimmune. This was significantly higher than the estimated prevalence in the overall population, approximately four times greater (31% versus 5–8%) [21]. This was supported by the higher observed occurrence of several autoimmune conditions, namely psoriasis (9% versus 3%), [22] RA (7% versus 1%), [23] inflammatory bowel disease (IBD) (4% versus 0.7%), [24] chronic urticaria (3% versus 0.5-1%), [25] lupus (2% versus 0.1%) [26] and psoriatic arthritis (2% versus 0.3%) [27, 28]. Prevalence of autoimmune conditions was similar between HAE-1, HAE-2, and HAE nC1-INHpatients, with non-statistical differences noted in thyroid disease (8% versus 3%), IBD (3% versus 7%), chronic urticaria (3% versus 7%), diabetes mellitus (1% versus 7%), and ankylosing spondylitis (0% versus 7%). In relation to gender differences in patient-reported autoimmune conditions, 34% of women in this study reported an autoimmune condition compared to 17% of men.

A higher prevalence of non-autoimmune comorbidities was reported by HAE patients in the survey with autoimmune conditions when compared to patients without autoimmune conditions. This difference was statistically significant with regards to hypertension (40% vs. 18%), sleeping disorder (40% vs. 11%), asthma (32% vs. 11%), irritable bowel syndrome (IBS) (29% vs. 9%), migraines/cluster headaches (32% vs. 7%), and arthritis (26% vs. 7%) (Table 3).

**Table 3:** Differences in comorbidities reported between respondents with and without autoimmune conditions

Comorbidities	Overall (*n* = 123)	Patients with autoimmune condition (*n* = 38)	Patients without autoimmune condition (*n* = 85)
Allergies to aeroallergens			
Allergies to pollen	27.6%	34.2%	24.7%
Allergies to pets/animal dander	23.6%	31.6%	20.0%
Allergies to dust mites	16.3%	23.7%	12.9%
Allergies to medicines	29.3%	36.8%	25.9%
Depression	27.6%	39.5%	22.4%
Hypertension*	24.4%	39.5%	17.7%
Sleeping disorder*^†^	19.5%	39.5%	10.6%
Asthma	17.1%	31.6%	10.6%
Hyperlipidemia	15.5%	23.7%	11.8%
Irritable bowel syndrome*	15.5%	29.0%	9.4%
Migraines/cluster headaches*	14.6%	31.6%	7.1%
Arthritis*	13.0%	26.3%	7.1%
Allergies to food	13.0%	15.8%	11.8%
Vision disorder	12.2%	21.1%	8.2%
Anemia	10.0%	13.2%	8.2%

*Indicates statistically significant difference in comorbidities reported between respondents with and without autoimmune conditions (*p* ≤ 0.05)

^†^Sleeping disorders included: insomnia, sleep apnea, restless leg syndrome, narcolepsy, etc

Patients with autoimmune conditions were found to have a less positive overall health status. Respondents without autoimmune conditions were more likely to have a very good to excellent state of health (53%), whereas only 18% of those with an autoimmune condition similarly reported having very good to excellent state of health. Respondents with an autoimmune condition were more likely to report being overweight compared to those without an autoimmune condition: obese (8% vs. 1%), overweight (50% vs. 39%), normal/healthy weight (40% vs. 57%). 32% of patients with autoimmune conditions reported having mobility issues, which is much higher than patients without autoimmune conditions (12%).

### Allergies and asthma

Overall, the numbers for patient-reported allergies are two times higher than that of the general population (54% vs. 27%^18^). Allergies were classified as allergies to medication (55% vs. 28%^18^), pollens (55% vs. 41%^18^) and pets/dander (44% vs. 29%^18^). The occurrence of these three allergies is significantly higher than that observed in the general population and were more commonly reported in patients with an autoimmune condition and among patients with HAE nC1-INH.

HAE patients reported a higher rate of asthma in the survey. Asthma was reported by 17% of respondents, which is significantly higher than the general population (8–11%) [29].

### HAE treatment initiation and evolution of comorbidities

**Fig. 3:**
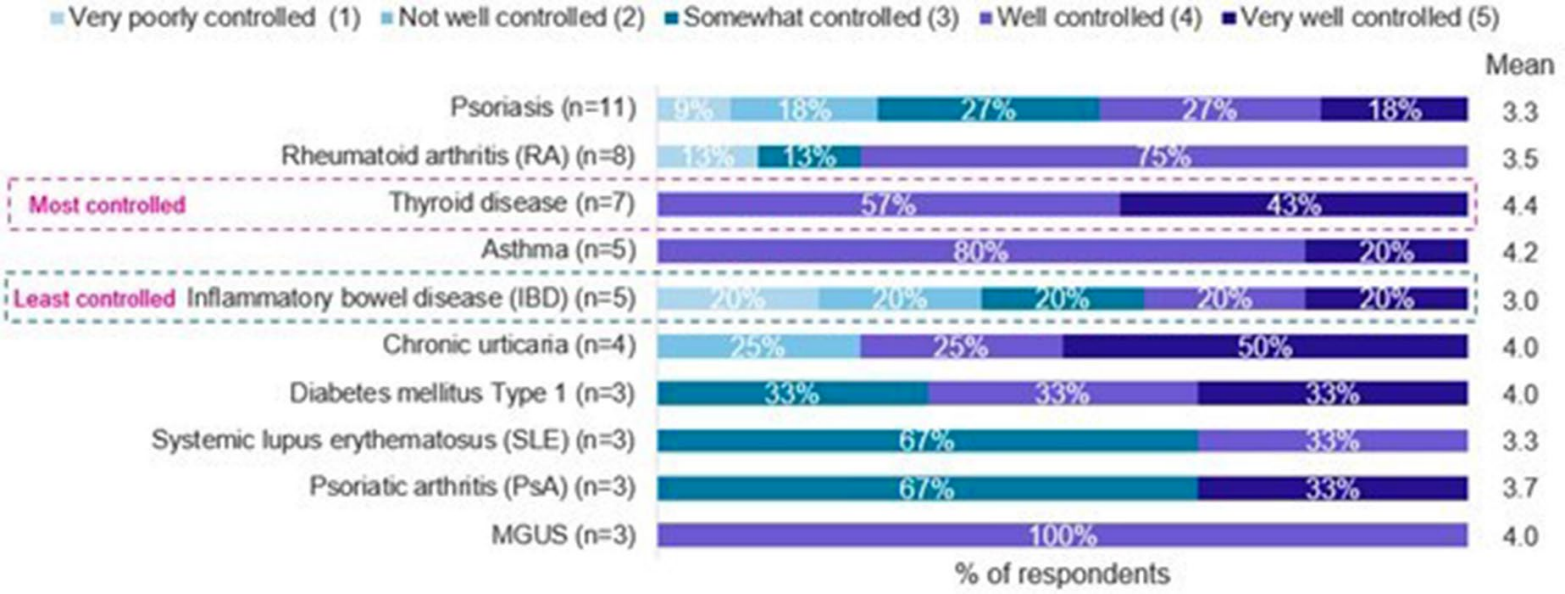
Current status and level of control of comorbidities varies by disease

In most cases, except for endocrine, nutritional, or metabolic conditions, patients were diagnosed with a comorbidity before their HAE diagnosis. The level of control of comorbidities varied by patient and by disease. For example, thyroid disease was reported as the most controlled and IBD was reported as the least controlled (Fig. 3). Overall, 27% of patients have seen an improvement in their given comorbidity since starting on HAE treatment, while 17% reported a worsening of their condition. Also, 63% of patients reported their current comorbidity condition as well to very well controlled, while only 16% of respondents reported poor to very poor controlled conditions.

**Fig. 4:**
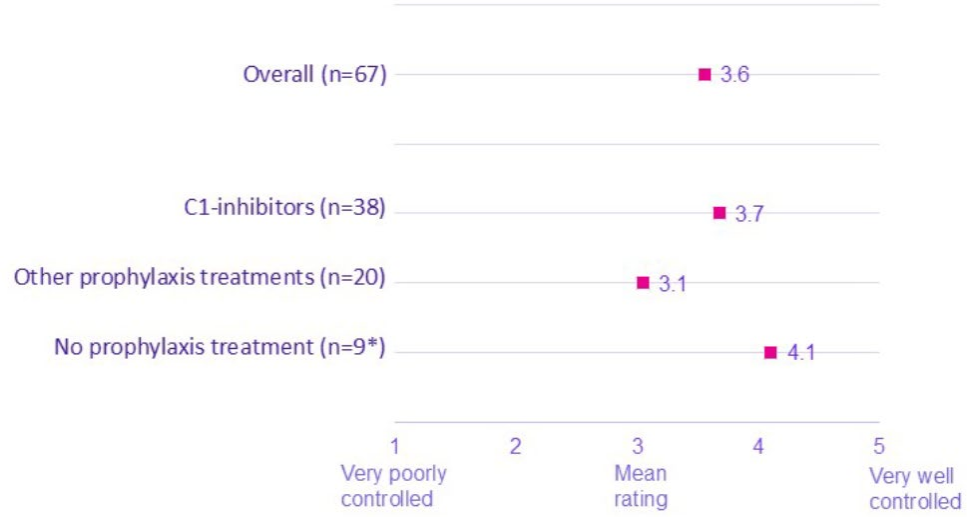
Current status of comorbidity by HAE prophylaxis taken. *Note: Low base. **Mean scores were significantly higher among patients currently on C1-INH compared to those on other HAE prophylaxis treatments (3.7 vs. 3.1). ***The study was not designed nor powered to detect a link between C1-inh and comorbidities. Other Prophylaxis treatments include: lanadelumab, danazol, tranexamic acid, icatibant, and other prophylaxis treatments

**Fig. 5:**
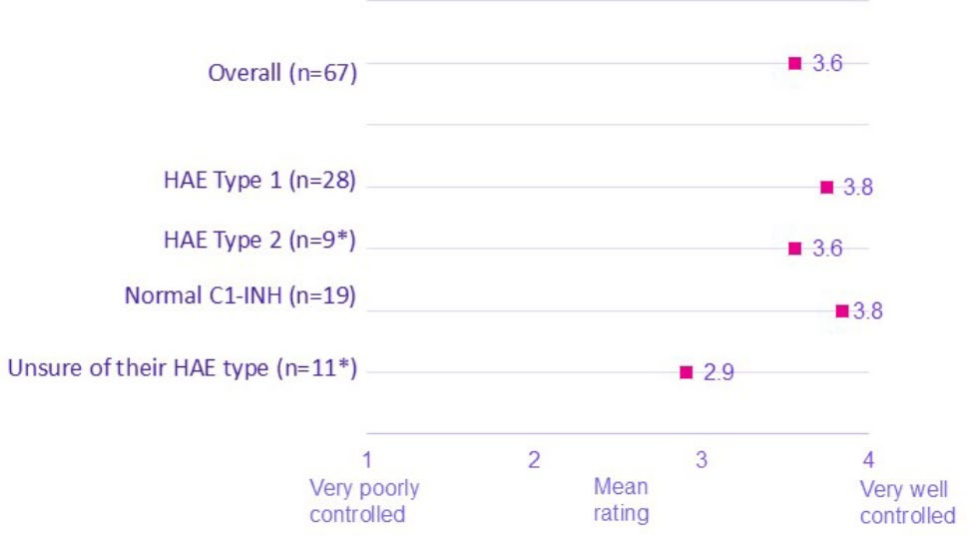
Current status of comorbidity by HAE Type. *Note: low base. **Mean scores were significantly higher among patients HAE-1 & HAE nC1-INH compared to those unsure of their HAE type. ***The study was not designed nor powered to detect a link between C1-inh and comorbidities

In particular, patients on C1-INH prophylaxis therapy (subcutaneous or intravenous pdC1inh) reported their comorbidity being better controlled compared to those on other prophylaxis treatments (lanadelumab, danazol, tranexamic acid, icatibant, and other). On a scale of 1 to 5 (1 being very poorly controlled and 5 being very well controlled), mean scores were significantly higher among patients currently on pdC1-INH compared to those on other HAE prophylaxis treatments (3.7 vs. 3.1). Patients with HAE-1 (3.8) and HAE nC1-INH (3.8) reported their comorbidity being better controlled compared to those with HAE-2 (3.6) or those who were unsure of their HAE type (2.9) (Figs. 4 and 5).

### Family history and predisposition to comorbidities

Patients also reported a family predisposition, with 45% of patients reporting a shared comorbidity with a family member, which is as high as 53% of HAE nC1-INH patients (most of these family members being a first degree relative, namely the mother). One third (30%) of these family members also have been diagnosed with HAE. This is as high as 40% of HAE nC1-INH patients that also have a relative with HAE.

### Hospitalization history due to comorbidities

Very few patients reported an unplanned emergency room (ER) visit or hospital stay due to their comorbidity in the previous 24 months. Only 12 reported unplanned ER visit or hospital stay for RA, arthritis, IBD, migraines, asthma, restless legs syndrome, and fibromyalgia (each represented by 1 respondent, requiring 1 to 2 ER visits). Some conditions required numerous ER visits and at least 1 hospitalization (represented by 1 patient each): allergies, chronic pancreatitis, mast cell activation syndrome, and eosinophilic asthma.

## Discussion

In this survey study, a high rate of comorbidities was observed, particularly with regard to autoimmune conditions, allergies, and asthma. This study was conducted to explore the link between HAE and comorbidities, as reported by patients, specifically in the Canadian setting. Canada has a national health care system and thus may have particularly good access to HAE prophylactic treatments compared to the rest of the world. Therefore, this study will enable comparisons to other countries, and similar to other studies based in other countries [2, 7], the results of this study suggest that HAE patients suffer from an increased risk of comorbidities when compared to the Canadian general population (4.3 versus 3.1 [30], respectively). Over two-thirds of respondents reported having at least one autoimmune condition (e.g., psoriasis, RA, inflammatory bowel disease, chronic urticaria, lupus, and psoriatic arthritis), allergy (e.g., medication, pollen, and pets/dander), or asthma, with many of the respondents reporting at least two of these conditions.

This study adds to published evidence from previous studies that show that HAE patients are more likely to have an autoimmune condition [7, 31, 32]. One of the explanations for this has been that the reduction of early complement components, specifically C4, in HAE patients, could affect the clearance of immune complexes, and immune regulation, which may in turn lead to autoimmunity [7, 33, 34]. The role of sex hormones in the pathogenesis of autoimmune diseases has been another possible explanation, particularly the influence of serum estrogen levels [33]. Notably, the pathophysiology of HAE nC1-INH is poorly understood but the role of sex hormones has been hypothesized, considering the higher prevalence of women living with HAE nC1-INH compared to men [33, 35].

Respondents with autoimmune conditions also suffered from more comorbidities overall than those without, which negatively impacted their quality of life and state of health, including increased mobility issues. While many of the mobility issues may be caused by swelling of the extremities in relation to HAE [36], they may also be a result of mobility issues associated with commonly reported HAE comorbidities, such as, RA and other arthritic conditions [37, 38]. Additionally, it should be noted that autoimmune diseases are more common in women, occurring at a rate of two to one compared to men [39]. Notably, 80.5% of patients in this study are women, and 34% of these women reported an autoimmune condition compared to 17% of men). This study was not designed to assess the gender-related differences in autoimmune conditions, however, this study still suggests that there is a higher proportion of patients with autoimmune disease in HAE patients, even when compared to the general population of women in Canada (31% versus 5–8%).^21^

Respondents reported a high rate of allergies, in particular to medications, pollen, pets/dander, dust mites, and food. However, allergies may be overestimated, as many patients with HAE may be diagnosed with allergies on their journey to being correctly diagnosed [40]. One survey specifically identified that misdiagnosis of HAE was among the top challenges reported by healthcare providers in the United States currently practicing allergy or immunology, and seeing at least one patient with HAE per year [41]. Similarly, another study looked at the impact of drug allergy labels among patients with HAE. The results of this study showed that misdiagnosed drug allergies are associated with delay in HAE diagnosis as well as increased likelihood of HAE attacks and rates of hospitalization, and interestingly, all patients in this study had their drug allergy labels removed after confirmatory drug tests [42]. Also, some studies have shown that autoimmune conditions may occur as a result of allergic inflammation [43–46]. The current study is consistent with the literature [47–50], in that respondents reported a delay in diagnosis and in treatment initiation. It is interesting to speculate that increased frequency of HAE attacks would not only worsen quality of life but may also increase the risk of developing comorbidities [2, 7, 13, 33, 51–56]. Therefore, it is essential to develop a better understanding of HAE, diagnostic delays, and the link to comorbidities.

Research has shown that C1-INH replacement as safe and effective in managing HAE [57–59]. One systematic review on randomized controlled trials concluded with C1-INH replacement therapy as an effective treatment for LTP in the management of HAE, with ≥ 90% reductions in HAE attack frequency achieved which could represent freedom from disease activity in clinical practice [57]. C1-INH replacement is best given at 60 u/kg, subcutaneous, twice weekly, and this study did not look at dose or adherence in this population. Understanding comorbidities in patients and how they respond to treatments is integral to our understanding of the treatment paradigm overall, because treatment for one condition may change the risk of another [7]. Limited research exists on the effects of C1-INH on comorbidities, though one study investigated the association between the type of HAE treatment (i.e., C1-INH replacement therapy for both on-demand and prophylaxis use) and comorbidities [31]. It is also possible that HAE patients with allergy and autoimmune comorbidities are more likely to require or benefit from pdC1inh replacement therapy compared to other HAE patients and that is why the cohort of patients studied had an increase in these conditions [8, 54]. For example, a small retrospective study by Farkas et al. [31]. observed that patients treated with pdC1-INH replacement therapy had lower number of visits for coexisting autoimmune disease compared to those treated with non-C1-INH treatments. While the current study was not powered to detect a link between C1-INH replacement and comorbidities, it did suggest that C1-INH replacement may better control comorbidities when compared to other prophylaxis, and future studies would benefit from being powered to explore this link further.

Limitations in this study were typical of survey study designs and patient reported data. One limitation was the potential for selection bias and survivorship bias as respondents were selected from the CSLB PLUS + PSP and it was possible that these patients are those that would typically report favourable response to pdC1inh treatment either given prophylactically or on demand. However, since patients were assessed on their comorbidities rather than their HAE disease control, the effect of selection and survivorship bias was limited, as these patients would likely have these comorbidities whether they were in this PSP program or not. Notably, neither C4 level nor C1 functional level were measured to assess whether patients were on an adequate LTP dose and adherence was not assessed. In addition, only 49% of the 85% on prophylactic pdC1-INH were taking subcutaneous pdC1-INH and thus 36% may have been on an inadequate dose of intravenous pdC1-INH or off-label subcutaneously. Selection bias was possible as patients with comorbidities may have been more likely to participate in this study compared to those without underlying comorbidities and recall bias may also have occurred among respondents as they had to remember details of diagnosis around comorbidities or their past experience with HAE, as patient medical charts were not accessible for this study. Regardless, the results of this study are consistent with conclusions of previously published studies [6–8].

Lastly, there was no control group in the analysis from the general population. Incidence of reported concomitant conditions were compared with prevalence within the Canadian population obtained through secondary research, whereby sources varied and thus were intended as indicators for comparison purposes only. Also, there were not enough patients to analyze by subgroups across all domains (i.e., HAE-1 and HAE-2 versus HAE nC1-INH versus general population). Future studies would benefit from the inclusion of a control group and the stratification of comorbidities and the types of comorbidities by HAE type. A challenge with studying rare diseases is the relatively small sample sizes to conduct subgroup analyses to elicit power in results that are sub-grouped. Future studies would also benefit from comparing patient reported comorbidities with their patient charts or medical records to verify the results. However, given that the results of this study are similar to previously published studies [6–8] and given the relatively high sample size for a rare disease study, this study is generalizable to the HAE population in Canada.

This study confirms that HAE patients suffer from more than just the symptoms of angioedema. HAE is a condition that appears to be associated with many comorbidities compared to the general population. In particular, these include an increased risk of autoimmune conditions, allergies, and asthma, contributing to a less positive overall health status. It is important to increase awareness among physicians who care for HAE patients on the relationship between HAE and comorbidities, to optimize the care of these patients. Furthermore, this study supports the need for robust registry data in the future to directly answer many of these questions.

## Data Availability

The data that support the findings of this study are not openly available due to reasons of sensitivity and are available from the corresponding author upon reasonable request, but restrictions apply to the availability of these data.

## Abbreviations

C1-INH: C1 esterase inhibitor
ER: Emergency room
HAE: Hereditary angioedema
HAE-1: HAE with deficit C1-inhibitor levels
HAE-2: HAE with dysfunctional C1-inhibitor
HAE nC1-INH: HAE with normal C1-inhibitor
IBD: Inflammatory bowel disease IBS: Irritable bowel syndrome
ICD: International Classification of Diseases
LTP: Long-term prophylaxis
pdC1inh: Plasma-derived C1-INH
PSP: Patient support program
RA: Rheumatoid arthritis
SD: Standard deviation
